# An n=1 Clinical Network Analysis of Symptoms and Treatment in Psychosis

**DOI:** 10.1371/journal.pone.0162811

**Published:** 2016-09-19

**Authors:** Maarten Bak, Marjan Drukker, Laila Hasmi, Jim van Os

**Affiliations:** 1 Department of Psychiatry and Psychology, School for Mental Health and Neuroscience, Maastricht University Medical Centre, Maastricht, The Netherlands; 2 King's College London, King's Health Partners, Department of Psychosis Studies, Institute of Psychiatry, London, United Kingdom; Istituto Superiore Di Sanita, ITALY

## Abstract

**Introduction:**

Dynamic relationships between the symptoms of psychosis can be shown in individual networks of psychopathology. In a single patient, data collected with the Experience Sampling Method (ESM–a method to construct intensive time series of experience and context) can be used to study lagged associations between symptoms in relation to illness severity and pharmacological treatment.

**Method:**

The patient completed, over the course of 1 year, for 4 days per week, 10 daily assessments scheduled randomly between 10 minutes and 3 hours apart. Five *a priori* selected symptoms were analysed: ‘hearing voices’, ‘down’, ‘relaxed’, ‘paranoia’ and ‘loss of control’. Regression analysis was performed including current level of one symptom as the dependent variable and all symptoms at the previous assessment (lag) as the independent variables. Resulting regression coefficients were printed in graphs representing a network of symptoms. Network graphs were generated for different levels of severity: stable, impending relapse and full relapse.

**Results:**

ESM data showed that symptoms varied intensely from moment to moment. Network representations showed meaningful relations between symptoms, e.g. ‘down’ and ‘paranoia’ fuelling each other, and ‘paranoia’ negatively impacting ‘relaxed’. During relapse, symptom levels as well as the level of clustering between symptoms markedly increased, indicating qualitative changes in the network. While ‘hearing voices’ was the most prominent symptom subjectively, the data suggested that a strategic focus on ‘paranoia’, as the most central symptom, had the potential to bring about changes affecting the whole network.

**Conclusion:**

Construction of intensive ESM time series in a single patient is feasible and informative, particularly if represented as a network, showing both quantitative and qualitative changes as a function of relapse.

## Introduction

The symptoms of mental illness have been represented as categories, dimensions and, more recently, mutually impacting states in a psychopathology network [1–3]. However, network models of psychopathology are difficult to study, as typical cross-sectional assessments of symptoms are not suitable to assess dynamic relationships between symptoms. Therefore, network models will profit from more fine-grained measures of psychopathology, represented as an intensive time series of experience and context, collected in the flow of daily life [4]. The assessment of symptoms as an intensive time series, randomly sampling experiences multiple times a day for a period of time, using the Experience Sampling Method (ESM), has become available for use in mental health practice, including psychosis [5, 6]. In this fashion, a unique dataset, allowing detailed ecologically valid examination of symptom interactions over time, can be collected at the level of the individual patient. ESM helps patients and professionals to gain insight in how symptoms impact on each other, and how treatments affect this pattern of dynamic interactions [7, 8]. As the number of connections between symptoms can become unmanageable, a focus on *a priori* selected key symptoms is required. Studies have shown that ESM symptom connections can be studied in relation to illness severity and clinical needs [9, 10] and can be represented by network graphs [11].

While ESM studies usually collect data for around 6 days, a more extended period is required for the monitoring of treatment effects [12].

### Aim

We present ESM data, representing an intensive time series of symptoms and context, in a single patient over a relatively protracted period of time (one year). Together with the patient, an *a priori* selected subset of symptoms was explored; ‘down’, ‘relaxed’, ‘paranoia’, ‘loss of control’ and ‘hearing voices’. The following questions were studied: (i) is it possible for a patient with a psychotic disorder to use ESM as a feedback tool for a year; (ii) to what degree do *a priori* selected symptoms co-occur and co-vary over the year; (iii) does the strength of the connections between symptoms depend on the within-person variation of illness severity? Given the focus on a single patient, no group-based hypotheses can be tested–the analyses presented are valid for a single patient. This study, however, also serves as proof-of-concept with implications for all patients treated for a mental disorder.

### Case Description

Miss A has been in treatment for severe psychotic experiences for twenty years. She is rarely free of symptoms, but their severity varies considerably over time. Variation in symptoms impacts her well-being, particularly when she relapses into a state in which symptoms are at their most severe. Psychopathology includes imperative hallucinations, paranoid delusions and low mood. Despite these challenges, she manages quite well, living independently and with a stable social network. She is a trained artist (painter) and passed her exams with distinction. In the last few years, she has taken up her profession as an artist again, making photographs and paintings, selling some of her work at expositions. Her case manager visits her once a week; she has monthly appointments with the psychiatrist.

In the late 1980s, she was admitted to a mental hospital with a first psychotic episode, followed by a long-term residential treatment programme. During this period, she attempted suicide several times. Ultimately, she became determined to reach the goal of independent living. This, and possibly a change in medication (clozapine) contributed to her personal recovery despite continuing psychopathology.

Miss A used several antipsychotics, mood stabilisers, antidepressants and benzodiazepines. Since the start of clozapine (about 14 years ago), a gradual but slow reduction of some other psychotropic medications became possible. She thus took the lead in the gradual discontinuation of benzodiazepines and promethazine, which was prescribed for insomnia and anxiety. She gradually learned to apply coping strategies to better deal with her symptoms.

Over the last 10 years, she and her psychiatrist agreed on a degree of self-management of the clozapine dose, allowing her to increase the dosage with 50 or 100 mg/day for a certain period in case of an impending relapse or a full relapse of her psychopathology, characterised by respectively a marked (100 mg increase) or moderate (50 mg increase) increase in symptom severity. She had found that with a temporary increase in the clozapine dose, symptoms would soon go down to the level where they were more manageable. As soon as she felt better, she would reduce the medication to the maintenance dose of 350 mg. In the last 4 years, Miss A had developed obesity and diabetes mellitus type 2 related to clozapine use.

During the ESM assessment period, Miss A was prescribed the following drugs: sulpiride 800 mg/day, clozapine 350 mg/day (with increases of 50 or 100mg as required), citalopram 20 mg / day, metformin 850 mg/day, omeprazole 40 mg/day and simvastatin 40 mg/day.

According to Miss A, her mother had experienced symptoms of psychosis too. She does not know her biological father. Miss A was raised by her mother’s sister, described as a callous woman, with whom she felt permanently unsafe. She describes a ‘Cinderella’ position in the family, her nieces being favoured whilst her needs were neglected. Her aunt passed away about 10 years ago.

Miss A hears voices, most often her aunt, giving negative feedback, telling her she is not good or urging her to commit suicide. Her self-esteem is low, as is her basic trust, resulting in periods of paranoia and low mood. Miss A experiences daily life events as stressful with ensuing feelings of exhaustion, doubt, loss of control and increased severity of hallucinations and paranoia. Although there is awareness of these vulnerabilities and recurrent sequences of events, she can neither predict nor control the fluctuations in intensity and severity. Miss A and her psychiatrist agreed to monitor her symptoms, and fluctuations thereof, over an extended period of time, using ESM, given user-reported evidence that intensive monitoring may help to gain control and achieve better adjustment [13, 14].

## Method

### Subject

The patient was Miss A, aged 46 years and diagnosed with schizophrenia, paranoid type, according to DSM-IV [15].

### Informed consent

The study was approved by the IRB of the Institute for Mental health Care Eindhoven and De Kempen (GGZE), Eindhoven, The Netherlands. The patient received oral and written information on the planned use of the data she collected and she signed an informed consent form. The duration of the study was for as long as the patient thought the self-monitoring procedure was helpful.

### ESM procedure

ESM is a random time-sampling self-assessment technique. The subject is signalled by a device ten times a day at random moments between 7.30 AM and 10.30 PM. Details on the choice of number of beeps per day and the choice of random time sampling are discussed elsewhere [16]. After each signal (a beep), the subject is asked to answer questions on current psychopathology like mood, convictions or hallucinations, as well as on context and appraisal of the present situation, using a mobile device. Subjects have 5 minutes to answers the question, as research has shown that larger lags impact validity [5]. Miss A used the device 4 days per week for around 12 months. Questions were in Dutch. ESM was usually done on the Monday, Wednesday, Friday and Saturday, although sometimes other days were used if this was more convenient.

### Assessment of psychopathology with ESM

ESM assesses experience and context with various items rated on 7-point Likert scales (‘1’ not at all *to* ‘7’ very). Some items index psychotic psychopathology, for example, “*I hear voices*”, as validated previously [5, 17–19]. Other items reflect positive affect or negative affect. For the present analyses, the following psychosis-related ESM items were used in the analyses: ‘*I hear voices’*, ‘*I feel suspicious’* and ‘*I feel I am losing control’*, as these were most important for the patient. In addition, two affective items, reflecting opposite poles, were selected: ‘*I feel down’*, and *‘I feel relaxed’*.

### Full relapse and impending relapse

‘Full relapse’ was defined as the collaborative decision to increase the clozapine dose to 450 mg/day. The decision to increase the dose to 400 mg/day because of a moderate increase in symptom severity will hereafter be referred to as ‘impending relapse’. Medication was reduced to the maintenance dose of 350 mg/day, as soon as Miss A felt her symptoms were less prominent and more manageable (hereafter: stable state).

### Statistical Analysis

Statistical analyses and graphical representations were performed using Microsoft Excel 2010, Stata 13 [20, 21] and R [22, 23].

First, in order to assess variation over time, mean daily severity levels of ‘hearing voices’, ‘loss of control’, ‘paranoia’ and mood (‘down’ and ‘relaxed’) were plotted. Second, network graphs were generated stratified by level of severity (stable state, impending relapse and full relapse). Five linear regression models were analysed with ‘down’, ‘loss of control’, ‘paranoia’, ‘hearing voices’, and ‘relaxed’ as dependent variables. Independent variables, for all models, were the lag (t-1) of the same 5 variables. One example of a regression model is:

$$Down=B0+B1\times lag\;{}^{\prime }down^{\prime }+B2\times lag\;{}^{\prime }loss\;of\;control^{\prime }+B3\times lag\;{}^{\prime }paranoia^{\prime }+B4\times {}^{\prime }laghearing\;voices^{\prime }+B5\times {}^{\prime }lag\;relaxed^{\prime }+time+e$$

In this model, time is used to de-trend the analyses [24]. As opposed to most analyses, all data pertain to a single subject. Therefore, data do not have a multilevel structure and can be analysed with standard linear regression techniques.

Using the qgraph command in R [25], each network graph included 25 regression coefficients obtained from the regression analyses above. Thus, the 25 regression coefficients express the strength of the connections. In addition, Excel and the qgraph package were used to calculate *indices of centrality*, to compare the strengths of the networks across the three states (stable, impending relapse, full relapse) in a descriptive fashion [26]. The *outward strength* is the sum of the connections from a specific node to all other nodes. The *inward strength* is the sum of the connections from all nodes to a specific node. *Node strength* is the sum of the inward strength and the outward strength [27]. In weighted directed networks, the inclusion of the self-loop (e.g. slope between ‘down’ at t-1 and ‘down’ at t) is crucial. As the self-loop therefore was included in both the *inward* and the *outward strength*, the self-loop was included twice in the node strength. *Closeness centrality* indicates how close a specific node is to the other nodes; it is defined as the inverse sum of the shortest distances to all other nodes from a specific node [28]. A closeness-central symptom is one that most likely affects other symptoms [29]. *Betweenness centrality* is a measure of the number of shortest paths that passes the node, and it indicates the global influence of the node throughout the network [3]. For all centrality measures, stronger values indicate stronger connections. More detailed information on centrality indices can be found elsewhere [11, 28].

## Results

### Feasibility of long-term ESM as a treatment tool

Miss A used the device for a year. She answered 943 beeps at 201 selected days (mean 4.7 beeps per day, sd = 1.49, range 1–9); that is 47% of all beeps on the selected days. Although questions were completed at 943 beeps, there were some partial missing data. Taking these into account resulted in a mean of 939 completed data points for the different ESM variables.

### Symptom level and day-level variation

During the ESM year, there were 4 full relapses (mean 17.3 days, range 4–25) and 2 impending relapses (mean 19.0 days, range 7–31). Symptom levels were progressively greater across the impending relapse and full relapse states (Table 1).

**Table 1:** Descriptives stratified by proxies of levels of severity (all range 1–7).

	*stable state*, n = 662		*impending relapse*, n = 158		*full relapse*, n = 119	
	mean	sd	Mean	Sd	Mean	sd
‘Down’	2.13	1.57	1.96	1.60	2.64	1.78^1^
‘Loss of control’	1.60	1.43	1.40	1.19	2.06	1.83^1^
‘Paranoia’	2.76	2.01	2.53	1.93	2.95	2.08
‘Hearing voices’	5.00	1.59	4.71^2^	1.59	4.78	1.63
‘Relaxed’	4.05	1.41	4.01	1.32	3.54	1.30^1^

^1^ In full relapse state, ‘down’ and ‘loss of control’ are significantly higher than in the stable state, whilst ‘relaxed’ is significantly lower.

^2^ In the impending relapse state, ‘hearing voices’ is significantly lower than in the stable state.

**Fig 1:**
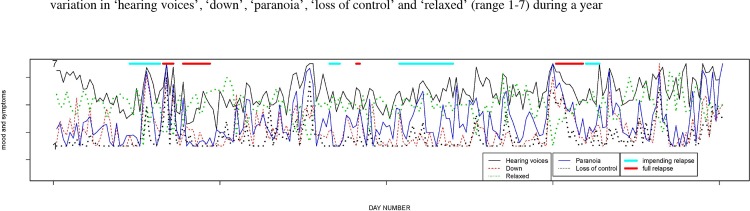
Network graph of five psychopathology items stratified by severity.

Generally, Miss A rated ‘hearing voices’ higher than ‘paranoia’, ‘down’ and ‘loss of control’ (Table 1). When inspecting day-level symptoms visually (Fig 1), ‘hearing voices’ varied considerably from maximum to moderately severe levels. However, day-level standard deviations of the symptoms did not differ significantly across the three states (data not shown). High levels of ‘down’ and low levels of ‘relaxed’ co-varied together, and vice-versa. In addition, levels of ‘down’ and ‘paranoia’ appeared to similarly co-vary. Generally, ‘loss of control’ was low. However, during periods of impending relapse, levels of ‘loss of control’ increased.

### Symptom Networks

**Fig 2:**
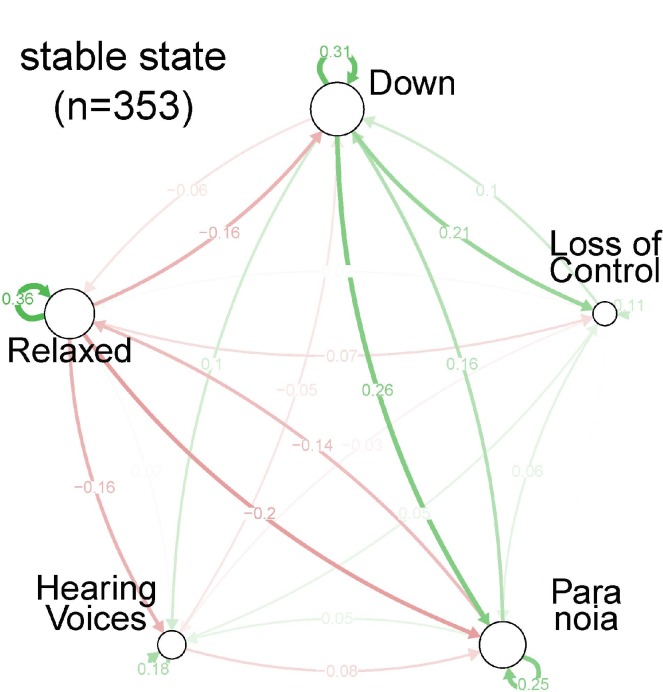
Centrality indices per symptom, based on Spearman partial correlation coefficients, for each of the three strata of severity: stable state.

**Fig 3:**
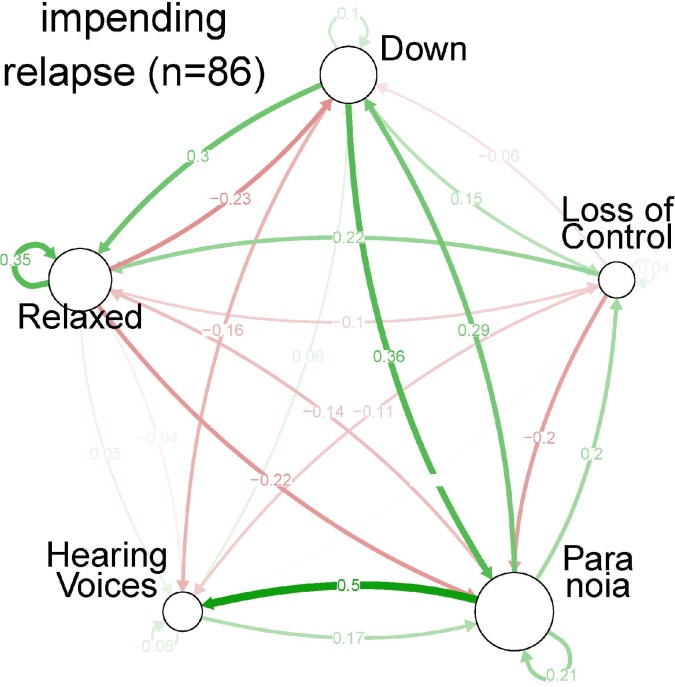
Centrality indices per symptom, based on Spearman partial correlation coefficients, for each of the three strata of severity: impending state.

**Fig 4:**
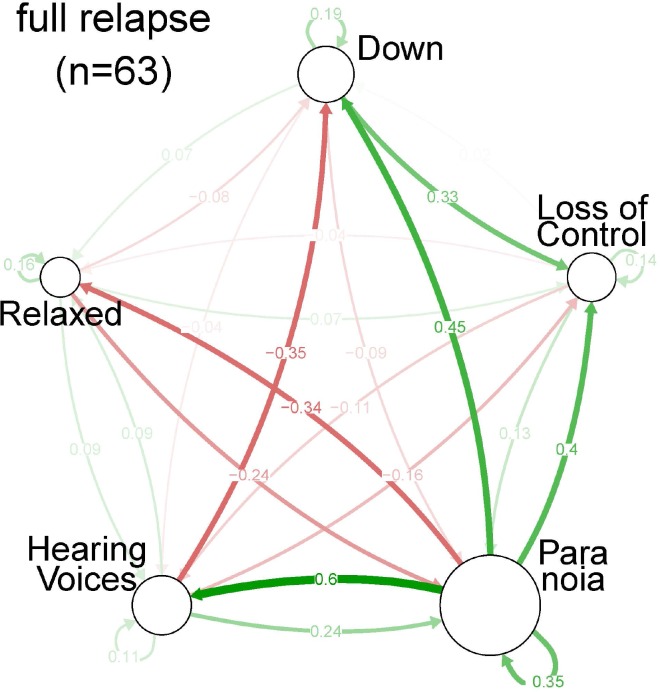
Centrality indices per symptom, based on Spearman partial correlation coefficients, for each of the three strata of severity: relapse state.

In the stable state, visual inspection showed a two-sided positive loop between ‘down’ and ‘paranoia’ (Fig 2, Fig 3 and Fig 4), indicating that these two symptoms mutually reinforced each other. In addition, a two-sided negative loop between ‘relaxed’ and ‘paranoia’ was visible (mutual reduction). ‘Hearing voices’ was only weakly connected with the other four symptoms; only ‘relaxed’ was moderately negatively connected with ‘hearing voices’ (B = -0.16). Finally, ‘down’ had a moderately (B = 0.21) positive connection with ‘loss of control’.

In the full relapse state, the strength of most connections increased (Fig 2, Fig 3, Fig 4 and Table 2). For example, the two-sided negative loop between ‘relaxed’ and ‘paranoia’ increased. The connection between ‘down’ and ‘paranoia’ was weaker, but the positive connection between ‘paranoia’ and both ‘hearing voices’ and ‘loss of control’ increased. In the full relapse state, ‘paranoia’ becomes the central node of the network.

**Table 2:** Centrality indices per symptom in the network, for each of the three strata of severity.

	Betweenness	Closeness	Inward degree	Outward degree	Node strength
**Stable state**					
‘Down’	5	0.032	0.78	0.92	1.70
‘Loss of control’	0	0.015	0.45	0.31	0.76
‘Paranoia’	4	0.026	0.87	0.62	1.48
‘Hearing Voices’	0	0.015	0.51	0.39	0.90
‘Relaxed’	1	0.036	0.61	0.98	1.59
**Impending relapse**					
‘Down’	2	0.056	0.74	1.07	1.81
‘Loss of control’	0	0.040	0.51	0.64	1.15
‘Paranoia’	7	0.058	1.16	1.34	2.49
‘Hearing Voices’	0	0.026	0.90	0.35	1.25
‘Relaxed’	0	0.039	1.05	0.95	2.00
**Full Relapse state**					
‘Down’	1	0.025	1.08	0.72	1.80
‘Loss of control’	3	0.027	1.12	0.44	1.56
‘Paranoia’	7	0.109	1.04	2.18	3.22
‘Hearing Voices’	0	0.050	0.95	0.95	1.90
‘Relaxed’	0	0.041	0.69	0.59	1.28

The Spearman correlations as suggested by reviewer #1

Although average levels of ‘hearing voices’ were relatively high, centrality indices for ‘hearing voices’ were relatively low (Table 2). In general, node strength increased across the impending and full relapse state and ‘paranoia’ showed a more central role. In impending relapse and full relapse state, connections between ‘paranoia’ and all other symptoms became stronger (outward degree of ‘paranoia’, inward degree of the other symptoms).

## Discussion

ESM in the clinical n = 1 situation appears to be feasible for a relatively long period of time. ESM data showed that symptoms varied intensely from moment to moment and that during subjectively defined relapse states, symptom levels as well as clustering of symptoms increased. Finally, in the most severe state, connections between symptoms increased to the degree that the patterns that were present during the stable state were no longer apparent, indicating qualitative changes in the network. The most severe symptom was not necessarily the most central in terms of network dynamics, as discussed below.

### ESM in treatment

Long term ESM assessment in a single patient was feasible and useful, yielding recognisable patterns of clinically relevant symptom interactions. Although only 47% of all potential beeps were rated, both patient and psychiatrist found the reported variability and clustering useful and interpretable. Visualisation of these patterns and connections provided additional information for both patient and psychiatrist. The information included daily variation in symptoms and impact of context or treatment on changes in symptoms over time. Earlier studies suggested that ESM is an appealing tool for the evaluation of medication effects, helping to fine-tune dosing [7, 13, 30–32]. The current study confirmed this pattern from a network and self-management perspective. ESM is less biased by mood, attention and memory problems that frequently occur in patients with psychotic symptoms, given the fact that it requires rating in the moment without retrospection [33]. Therefore, not only for Miss A but also for other patients, a more accurate and personalised treatment plan may be developed using prolonged ESM assessment including momentary variation of psychopathology [6]. The data suggest that clinical network analysis yields insights about underlying symptom-symptom and symptom-context dynamics–beyond the usual severity scores–that are useful to both patient and clinician [4]. In addition, clinical network analysis can be used to uniquely assess the impact of pharmacological and non-pharmacological treatment at the level of how symptoms impact on each other. Clinical network analysis thus may be useful when starting medication, changing medication or when tapering off medication [13, 32, 34]. In addition, empowering the patient to collect his own diagnostic and treatment evaluation data aids shared decision-making in clinical practice and enhances ‘ownership’ of the clinical process. This may result in reduced medication use and less unwanted side effects. Discussing ESM results with a patient offers clues as to why and when symptoms vary, given certain stressors and contexts, with clues for protective mechanisms or coping strategies [35].

### Symptom variability

During the one-year follow-up, the 5 *a priori* selected symptoms varied considerably. Visual inspection of Fig 1 showed stronger fluctuations of ‘hearing voices’, ‘paranoia’ and ‘down’ in periods of impending relapse and full relapse. In a previous report, increased levels of symptom severity were associated with the subjective sensation of ‘loss of control’ at the group level [36]. Miss A’s choice to increase clozapine dosage may reflect periods of more psychopathology, in terms of severity or impact on daily life, resulting in a subjective feeling of loss of control. She learned over time that a temporary increase in clozapine dosage helps her to regain control.

### Network analysis

During the stable state, ‘down’ and ‘paranoia’ were most strongly interconnected. Virtually all connections grew stronger in the impending relapse and full relapse state. This suggested that in relapse states, symptoms were more connected, as observed previously in other samples [19]. Centrality indices showed a shift towards ‘paranoia’ and to a lesser extent ‘hearing voices’, during relapse. Betweenness underlined the crucial role of paranoia in the network, whereas ‘hearing voices’ had a less important central role. These data suggest that while ‘hearing voices’ is the most prominent symptom subjectively (Table 1); a strategic focus on ‘paranoia’ may bring about changes that affect other symptoms in the network (Table 2). However, this type of clinical reasoning will only hold if one assumes that connections between symptoms reflect causal relationships, which is uncertain. While two symptoms may impact on each other causally, their connections may also reflect a higher order alteration driving variation in both.

The impending relapse state showed symptom connection strengths that were in between those observed in the stable and the full relapse state, validating the changes in medication dose that formed the basis for the definition of full relapse and impending relapse. In the impending relapse state, centrality indices were comparable to those in the full relapse state. Similarly, ‘paranoia’ was the most central symptom although less prominently than in the full relapse state.

The data additionally suggest that patients can self-manage and self-monitor their medication use, within certain boundaries. This is in agreement with emerging evidence in other areas in medicine [37]. In addition, the increased level of clustering of symptoms during relapse suggests that greater levels of clustering of these symptoms are indicative of clinical need and dysfunction, as shown previously [9].

### Binary diagnosis in relation to network analysis

The notion that mental disorders are dichotomous unidimensional entities defined by a set of criteria may be incomplete. Correlated symptoms are distributed over a continuum of severity; not all persons with some degree of expression of an extended phenotype meeting diagnostic criteria [27, 38]. Given strong trans diagnostic correlations between symptoms, classification of mental symptoms into mental diagnoses results in a considerable overlap between psychiatric diagnoses at various levels [39]. Network analysis of extended phenotypic expression of symptoms may confer added validity to representations of mental disorder [3, 4, 40]. In addition, network analysis shows that symptoms cluster into patterns [27, 28, 40–42]. Cross-sectional network analysis can be seen as an improved factor analysis or principal component analysis, visualising connections between symptoms two-dimensionally [3]. The present paper, in agreement with previous work [27, 28] generated networks including a time component with ESM data, provides a solution for the problem of temporal under-sampling of psychopathology in cross-sectional network analysis. ESM has the advantage of building intensive time series of experiences as emerging in the flow of daily life, which is not the case in the model using cross-sectional measures of psychopathology based on retrospection and interpretation [40]. Despite studying a limited set of symptoms, the present analysis including the time factor shows that networks are dynamic; the clustering of symptoms changes depending on external factors. Network analysis, therefore, can complement the practice of categorical classification.

### Methodological issues

To our knowledge, this is the first study to use long-term ESM data collected in a single patient with a psychotic disorder, showing real life fluctuations in symptoms and the impact of symptom severity. MB is treating psychiatrist of Miss A. MB and Miss A discussed the raw data four times during the period of data collection.

When interpreting the results, some limitations must be considered. First, generalizability in the strict sense is limited. The graphs in the present paper are specific for this patient. On the other hand, the present results did show these graphs yield information that is interpretable and useful–and as such may be considered generalizable. Each patient has unique characteristics and we believe that more attention to personal symptom variation and patterns of connectivity is helpful in developing a personalized treatment plan. In addition, the present results can be replicated in other patients with the same diagnosis, allowing for meta-analytic identification of group effects.

Second, although Miss A used the ESM device for about a year, it was used only four days a week. ESM data covered a period of 201 days, collected over a period of one year. Thus, hypothetically, the data could have included a maximum of 2010 beeps with completed data. Instead, Miss A filled in questions at 943 beeps, which, taking into account partial missing data, resulted in a mean of 939 completed data points for the different ESM variables. When completing an ESM time series, it is unavoidable that the person misses beeps, regardless of the presence of mental illness [16]. Missing beeps may be unavoidable, for example in the morning when the participant is asleep or in the afternoon when the participant is taking a nap [43, 44]. The ESM sampling frame therefore oversamples up to 10 times per day to compensate. Furthermore, a higher proportion of missing beeps in the present paper may be expected given that the patient was diagnosed with psychotic disorder and used ESM for nearly a year. Previous work on ESM in patients with psychotic disorder has established that validity is preserved if at least 30% of beeps are completed [6]. Previous work on ESM in patients with psychotic disorder has established that validity is preserved if at least 30% of beeps are completed [6]. Therefore, the fact that less than 50% of beeps were completed is unlikely to affect the validity of the results. Nevertheless, the information obtained was substantial. In addition, the number of valid beeps per day only slightly differs across stable (4.7; range 1–8), impending relapse (4.4 range 1–9) and full relapse states (5.0; range 2–8; F = 1.08, p = 0.34). This suggests that data are not biased because of oversampling or under sampling during periods of relapse.

Third, although regression analysis is the tool to generate networks [28], results can be instable in the sense that multiple models with different coefficients can have similar fit. The use of Spearman partial correlations has been advocated to get stable results [45]. S1 Table and S1 Fig, S2 Fig and S3 Fig presents networks of Spearman partial correlations from a sensitivity analysis using the ppcor package in R and the accompanying centrality measures [46]. In stable and impending relapse states, results were essentially similar; only the connection between down and paranoia was less strong in the Spearman network. In full relapse state, the loop between down and relaxed was stronger. On the other hand, ‘betweenness’ was different between the original analysis and the sensitivity analyses (see S1 Table and S1 Fig, S2 Fig and S3 Fig).

Furthermore, the importance and clinical relevance of betweenness centrality and shortest paths in weighted networks of symptoms, as presented in the current paper, is uncertain. While in unweighted networks shortest paths are easy to define, in weighted directional networks, several paths may have approximately the same weight, making it difficult to identify a single shortest path. This is illustrated by the large differences in betweenness between the original analyses and the sensitivity analyses, while connection strength and other centrality measures were similar. In addition, our regression analyses to obtain the strength of the connections included all symptoms (at t-1) simultaneously so that all connections only represent direct paths. In other words, all paths are important, not only the shortest. Therefore, the advantage of being located on the shortest path (betweenness) is limited.

Finally, the 5 symptoms chosen *a priori* for analysis are 5 key symptoms, identified jointly by Miss A and her psychiatrist (MB). It is a reduction of reality and represents a small proportion of the symptoms available in ESM. Although this simplification makes it possible to better identify the network dynamics of included symptoms, results may differ depending on which symptoms are chosen for inclusion in the analyses.

### Conclusions and recommendations

Prolonged use of ESM self-monitoring is feasible in at least some patients diagnosed with psychotic disorder. Graphs of data pertaining to a single individual can be scrutinized in order to identify patterns that can be helpful in treatment. Although Miss A and her psychiatrist discussed raw data only, in the future, patients may benefit from more immediate ‘on the go’ graphs based on recent input. Network analysis shows that relapse in this patient coincided with a recognisable shift in symptoms. ESM, therefore, offers the possibility for accurate and personalised interventions in patients with mental disorder, including psychosis. It is another tool that can aide in understanding a patient’s symptoms, how symptoms interact with each other and how symptoms are influenced by context. ESM data as collected by Miss A may assist in predicting relapse and other prognostic measures, facilitating the formulation of tailor-made interventions. However, the interaction between symptoms in psychotic disorders is complicated, and more work is required on how ESM n = 1 clinical network analysis can assist clinical practice.

## Supporting Information

S1 Fig Network graph of five psychopathology, based on Spearman partial correlation coefficients, items stratified by severity: stable state. (TIFF)

S2 Fig Network graph of five psychopathology, based on Spearman partial correlation coefficients, items stratified by severity: impending state. (TIFF)

S3 Fig Network graph of five psychopathology, based on Spearman partial correlation coefficients, items stratified by severity: relapse state. (TIFF)

S1 Table Centrality indices per symptom, based on Spearman partial correlation coefficients, for each of the three strata of severity. (DOCX)

## References

[1] Borsboom D, Cramer AO. Network analysis: an integrative approach to the structure of psychopathology. Annu Rev Clin Psychol. 2013;9:91–121. 10.1146/annurev-clinpsy-050212-185608 23537483

[2] Kendler KS, Zachar P, Craver C. What kinds of things are psychiatric disorders? Psychological medicine. 2011;41(6):1143–50. 10.1017/S0033291710001844 20860872

[3] Goekoop R, Goekoop JG. A network view on psychiatric disorders: network clusters of symptoms as elementary syndromes of psychopathology. PLoS One. 2014;9(11):e112734 10.1371/journal.pone.0112734 25427156 PMC4245101

[4] van Os J, Delespaul P, Wigman J, Myin-Germeys I, Wichers M. Beyond DSM and ICD: introducing "precision diagnosis" for psychiatry using momentary assessment technology. World Psychiatry. 2013;12(2):113–7. 10.1002/wps.20046 23737412 PMC3683255

[5] Delespaul PA, deVries MW. The daily life of ambulatory chronic mental patients. J Nerv Ment Dis. 1987;175(9):537–44. . 3655779 10.1097/00005053-198709000-00005

[6] Delespaul PAEG. Assessing Schizophrenia in Daily Life: The Experience Sampling Method [PhD Thesis]. Maastricht: Maastricht University; 1995.

[7] Kramer I, Simons CJ, Hartmann JA, Menne-Lothmann C, Viechtbauer W, Peeters F, et al A therapeutic application of the experience sampling method in the treatment of depression: a randomized controlled trial. World Psychiatry. 2014;13(1):68–77. 10.1002/wps.20090 24497255 PMC3918026

[8] Myin-Germeys I, Oorschot M, Collip D, Lataster J, Delespaul P, van Os J. Experience sampling research in psychopathology: opening the black box of daily life. Psychological medicine. 2009:1–15. . 19215626 10.1017/S0033291708004947

[9] van Os J, Lataster T, Delespaul P, Wichers M, Myin-Germeys I. Evidence that a psychopathology interactome has diagnostic value, predicting clinical needs: an experience sampling study. PLoS One. 2014;9(1):e86652 10.1371/journal.pone.0086652 24466189 PMC3900579

[10] Kramer I, Simons CJ, Wigman JT, Collip D, Jacobs N, Derom C, et al Time-lagged moment-to-moment interplay between negative affect and paranoia: new insights in the affective pathway to psychosis. Schizophr Bull. 2014;40(2):278–86. 10.1093/schbul/sbs194 23407984 PMC3932075

[11] Wichers M. The dynamic nature of depression: a new micro-level perspective of mental disorder that meets current challenges. Psychol Med. 2014;44(7):1349–60. 10.1017/S0033291713001979 23942140

[12] Wichers M, Simons CJ, Kramer IM, Hartmann JA, Lothmann C, Myin-Germeys I, et al Momentary assessment technology as a tool to help patients with depression help themselves. Acta Psychiatr Scand. 2011;124(4):262–72. Epub 2011/08/16. 10.1111/j.1600-0447.2011.01749.x 21838742

[13] Bos FM, Schoevers RA, Aan Het Rot M. Experience sampling and ecological momentary assessment studies in psychopharmacology: A systematic review. Eur Neuropsychopharmacol. 2015 10.1016/j.euroneuro.2015.08.008 . 26336868

[14] Groot PC. Patients can diagnose too: How continuous self-assessment aids diagnosis of, and recovery from, depression. J Ment Health. 2010;19(4):352–62. 10.3109/09638237.2010.494188 20636115

[15] American Psychiatric Association A. DSM-IV: Diagnostic and Statistical Manual of Mental Disorders. Washinton DC.: APA.; 1994.

[16] Verhagen SJ, Hasmi L, Drukker M, van Os J, Delespaul PA. Use of the experience sampling method in the context of clinical trials. Evid Based Ment Health. 2016;19(3):86–9. 10.1136/ebmental-2016-102418 27443678 PMC5040762

[17] Delespaul P, Bak M., Van Os J. Handleiding Maastrichtse Psychoseprotocol. 2e edition ed. Maastricht: Maastricht University; 2002.

[18] Bak M, Myin-Germeys I, Delespaul P, Vollebergh W, De Graaf R, Van Os J. Do different psychotic experiences differentially predict need for care in the general population? Comprehensive Psychiatry. 2005;46:192–9. 16021589 10.1016/j.comppsych.2004.08.003

[19] Wigman JT, Collip D, Wichers M, Delespaul P, Derom C, Thiery E, et al Altered transfer of momentary mental states (ATOMS) as the basic unit of psychosis liability in interaction with environment and emotions. PLoS One. 2013;8(2):e54653 10.1371/journal.pone.0054653 23457452 PMC3574136

[20] StataCorp. Stata Statistical Software. 11 ed. College Station, Texas: Stata Corporation; 2009.

[21] Statacorp. Statistical Software: release 11.: College Station, TX: Stata Corporation; 2009.

[22] R Core Team. R: A Language and Environment for Statistical Computing. In: Computing RFfS, editor. Vienna, Austria 2013.

[23] Team. RC. R: A Language and Environment for Statistical Computing. Vienna, Austria: Computing RFfS; 2013.

[24] Wang LP, Maxwell SE. On disaggregating between-person and within-person effects with longitudinal data using multilevel models. Psychol Methods. 2015;20(1):63–83. 10.1037/met0000030 25822206

[25] Epskamp S, Cramer AO, Waldorp LJ, Schmittmann VD, Borsboom D. Network visualizations of relationships in psychometric data. Journal of Statistical Software. 2012;48(4):1–18.

[26] Opsahl T, Agneessens F, Skvoretz J. Node centrality in weighted networks: Generalizing degree and shortest paths. Social Networks. 2010;32:245–51.

[27] Wigman JT, van Os J, Borsboom D, Wardenaar KJ, Epskamp S, Klippel A, et al Exploring the underlying structure of mental disorders: cross-diagnostic differences and similarities from a network perspective using both a top-down and a bottom-up approach. Psychological medicine. 2015:1–13. 10.1017/S0033291715000331 . 25804221

[28] Bringmann LF, Vissers N, Wichers M, Geschwind N, Kuppens P, Peeters F, et al A network approach to psychopathology: new insights into clinical longitudinal data. PLoS One. 2013;8(4):e60188 10.1371/journal.pone.0060188 23593171 PMC3617177

[29] Costantini M, Epskamp S, Borsboom D, Perugini M, Mottus R, Waldorp LJ, et al State of the aRt personlaity research: A tutorial on network analysis of persality data in R. Journal of Research in Personlaity. in press.

[30] Lataster J, Myin-Germeys I, Wichers M, Delespaul PA, van Os J, Bak M. Psychotic exacerbation and emotional dampening in the daily life of patients with schizophrenia switched to aripiprazole therapy: a collection of standardized case reports. Ther Adv Psychopharmacol. 2011;1(5):145–51. 10.1177/2045125311419552 23983939 PMC3736906

[31] Lataster J, van Os J, de Haan L, Thewissen V, Bak M, Lataster T, et al Emotional experience and estimates of D2 receptor occupancy in psychotic patients treated with haloperidol, risperidone, or olanzapine: an experience sampling study. J Clin Psychiatry. 2011;72(10):1397–404. Epub 2011/01/07. 10.4088/JCP.09m05466yel 21208588

[32] Wichers M, Groot PC, Psychosystems ESMGEWSG. Critical Slowing Down as a Personalized Early Warning Signal for Depression. Psychother Psychosom. 2016;85(2):114–6. 10.1159/000441458 26821231

[33] Blum LH, Vakhrusheva J, Saperstein A, Khan S, Chang RW, Hansen MC, et al Depressed mood in individuals with schizophrenia: A comparison of retrospective and real-time measures. Psychiatry research. 2015;227(2–3):318–23. 10.1016/j.psychres.2015.03.008 25895490 PMC4430399

[34] van Os J, Delespaul P, Barge D, Bakker RP. Testing an mHealth momentary assessment Routine Outcome Monitoring application: a focus on restoration of daily life positive mood states. PLoS One. 2014;9(12):e115254 10.1371/journal.pone.0115254 25513813 PMC4267819

[35] Lardinois M, Myin-Germeys I, Bak M, Mengelers R, van Os J, Delespaul PA. The dynamics of symptomatic and non-symptomatic coping with psychotic symptoms in the flow of daily life. Acta Psychiatr Scand. 2007;116(1):71–5. . 17559603 10.1111/j.1600-0447.2007.01022.x

[36] Declerck CH, Boone C, De Brabander B. On feeling in control: a biological theory for individual differences in control perception. Brain and cognition. 2006;62(2):143–76. . 16806623 10.1016/j.bandc.2006.04.004

[37] McManus RJ, Mant J, Haque MS, Bray EP, Bryan S, Greenfield SM, et al Effect of self-monitoring and medication self-titration on systolic blood pressure in hypertensive patients at high risk of cardiovascular disease: the TASMIN-SR randomized clinical trial. JAMA. 2014;312(8):799–808. 10.1001/jama.2014.10057 25157723

[38] van Os J, Linscott RJ, Myin-Germeys I, Delespaul P, Krabbendam L. A systematic review and meta-analysis of the psychosis continuum: evidence for a psychosis proneness-persistence-impairment model of psychotic disorder. Psychol Med. 2009;39(2):179–95. Epub 2008/07/09. 10.1017/S0033291708003814 18606047

[39] van Wijngaarden-Cremers PJ, van Deurzen P, Oosterling I, Groen W, Langen M, Lagro-Janssen AL, et al [A fresh look at psychiatric disorders]. Tijdschrift voor psychiatrie. 2014;56(10):670–9. . 25327349

[40] Borsboom D, Cramer AO, Schmittmann VD, Epskamp S, Waldorp LJ. The small world of psychopathology. PLoS One. 2011;6(11):e27407 Epub 2011/11/25. 10.1371/journal.pone.0027407 22114671 PMC3219664

[41] Cramer AO, Waldorp LJ, van der Maas HL, Borsboom D. Comorbidity: a network perspective. Behav Brain Sci. 2010;33(2–3):137–50; discussion 50–93. 10.1017/S0140525X09991567 20584369

[42] Goekoop R, Goekoop JG. [Network clusters of symptoms as elementary syndromes of psychopathology: implications for clinical practice]. Tijdschrift voor psychiatrie. 2016;58(1):38–47. . 26779754

[43] Johnson EI, Grondin O, Barrault M, Faytout M, Helbig S, Husky M, et al Computerized ambulatory monitoring in psychiatry: a multi-site collaborative study of acceptability, compliance, and reactivity. International journal of methods in psychiatric research. 2009;18(1):48–57. 10.1002/mpr.276 19195050 PMC6878313

[44] Silvia PJ, Kwapil TR, Eddington K.M., Brown LH. Missed beeps and missing data: Dispositional and situational predictors of nonrespons in experience sampling Social cience and computer review. 2013;31(4):471–81.

[45] de la Fuente A, Bing N, Hoeschele I, Mendes P. Discovery of meaningful associations in genomic data using partial correlation coefficients. Bioinformatics. 2004;20(18):3565–74. 10.1093/bioinformatics/bth445 . 15284096

[46] Kim S. ppcor: An R Package for a Fast Calculation to Semi-partial Correlation Coefficients. Commun Stat Appl Methods. 2015;22(6):665–74. 10.5351/CSAM.2015.22.6.665 26688802 PMC4681537

