# Correction: An n=1 Clinical Network Analysis of Symptoms and Treatment in Psychosis

**DOI:** 10.1371/journal.pone.0165762

**Published:** 2016-10-27

**Authors:** Maarten Bak, Marjan Drukker, Laila Hasmi, Jim van Os

There are errors in the captions for Figs 2, 3 and 4 of the manuscript. Please see the complete, correct Figs 2, 3 and 4 captions here.

**Fig 2:**
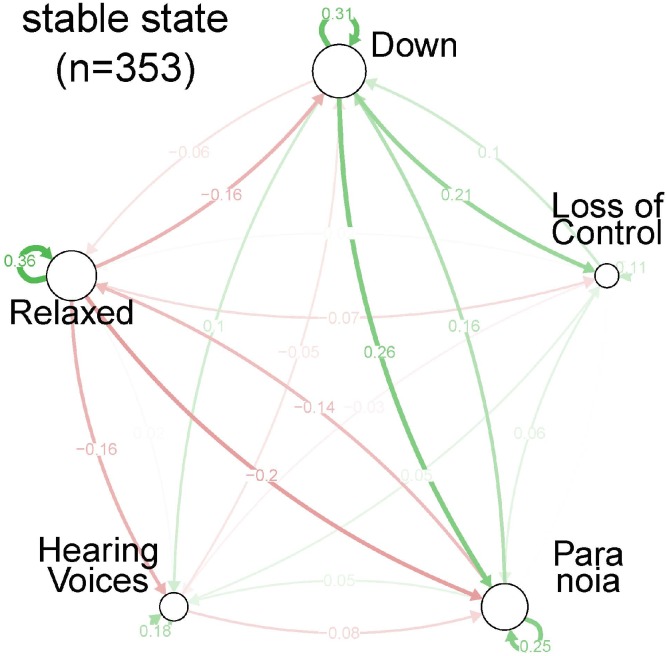
Centrality indices per symptom, for each of the three strata of severity: stable state.

**Fig 3:**
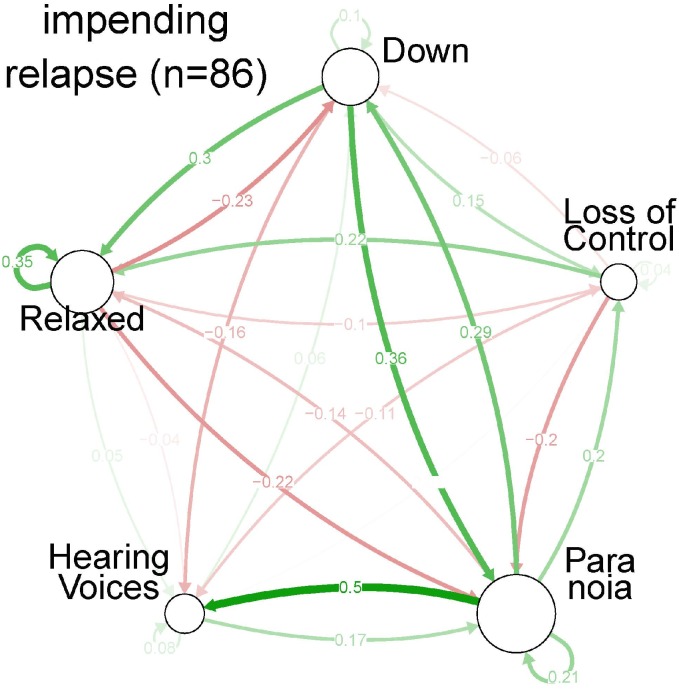
Centrality indices per symptom, for each of the three strata of severity: impending state.

**Fig 4:**
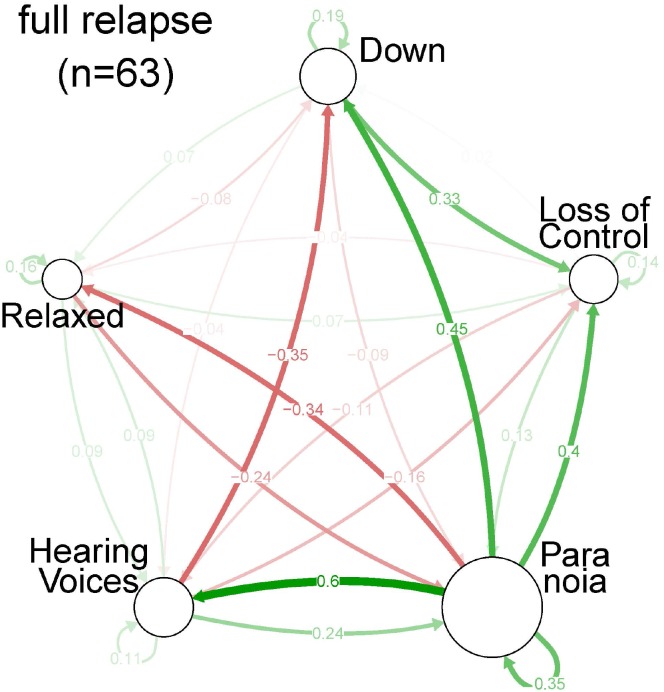
Centrality indices per symptom, for each of the three strata of severity: relapse state.
