# Supplementary figures and images for: An n=1 Clinical Network Analysis of Symptoms and Treatment in Psychosis
Source: PLoS One. 2016 Sep 19;11(9):e0162811. doi: 10.1371/journal.pone.0162811 (PMC5028060; doi:10.1371/journal.pone.0162811)

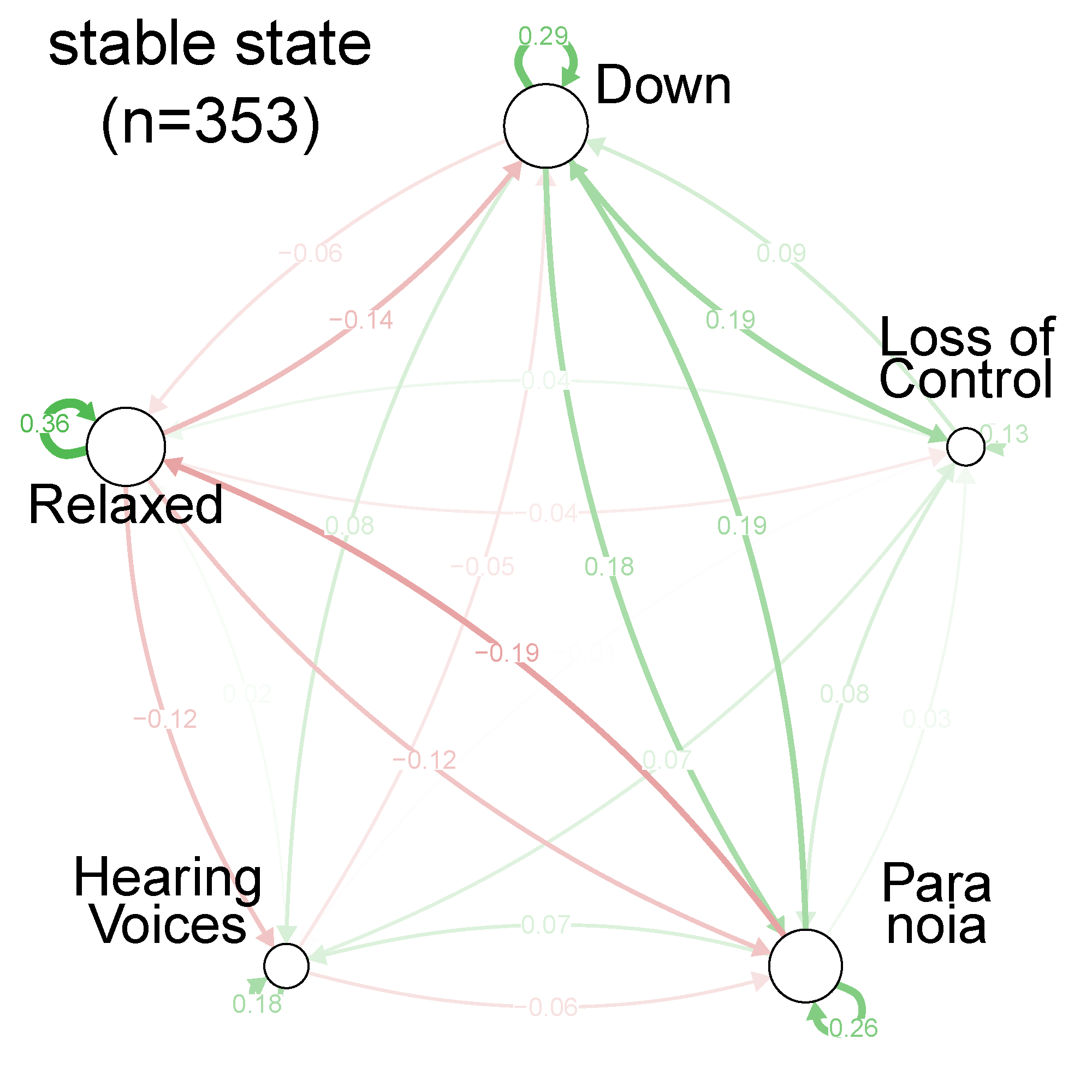

Supplement: S1 Fig — (TIFF) [file pone.0162811.s001.tiff]

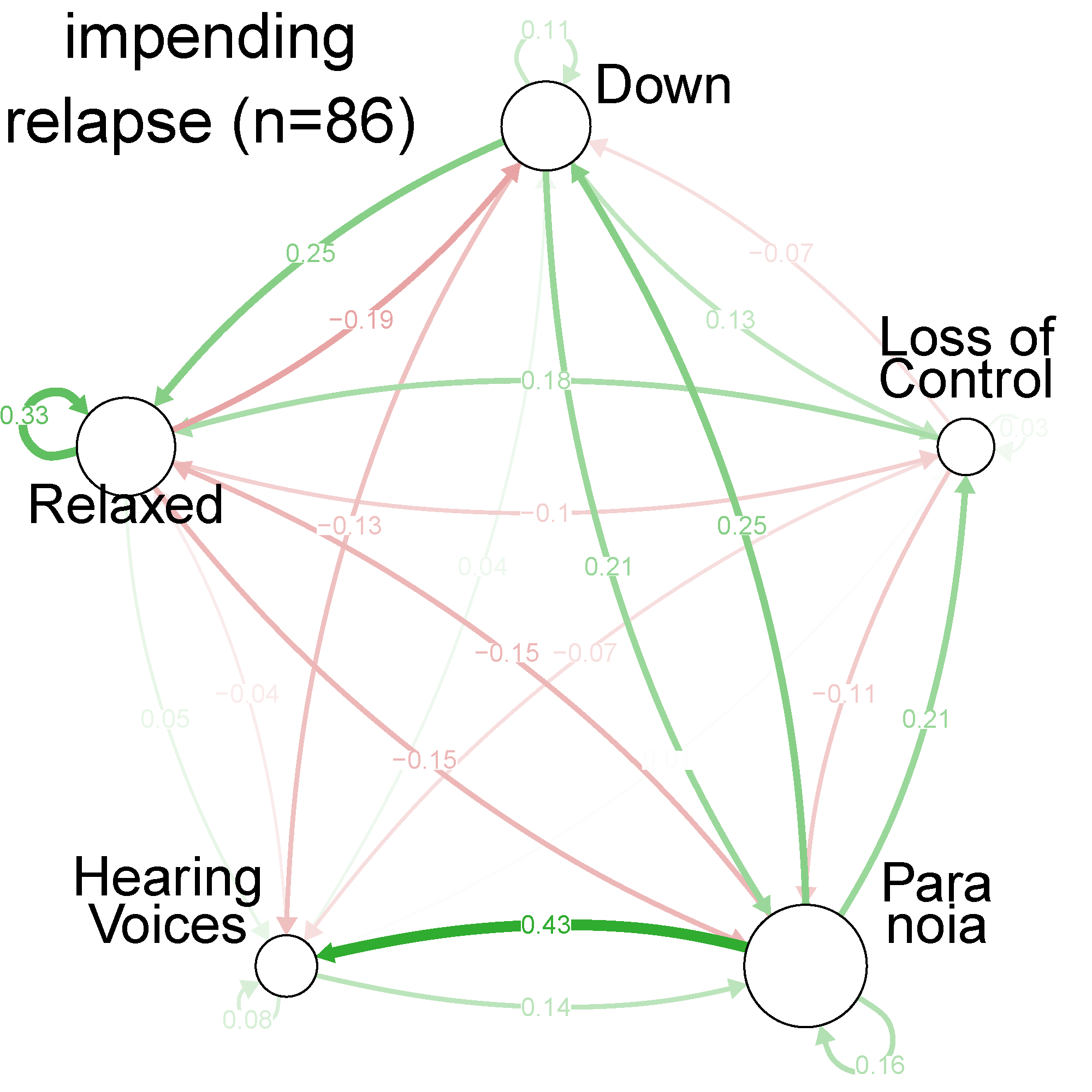

Supplement: S2 Fig — (TIFF) [file pone.0162811.s002.tiff]

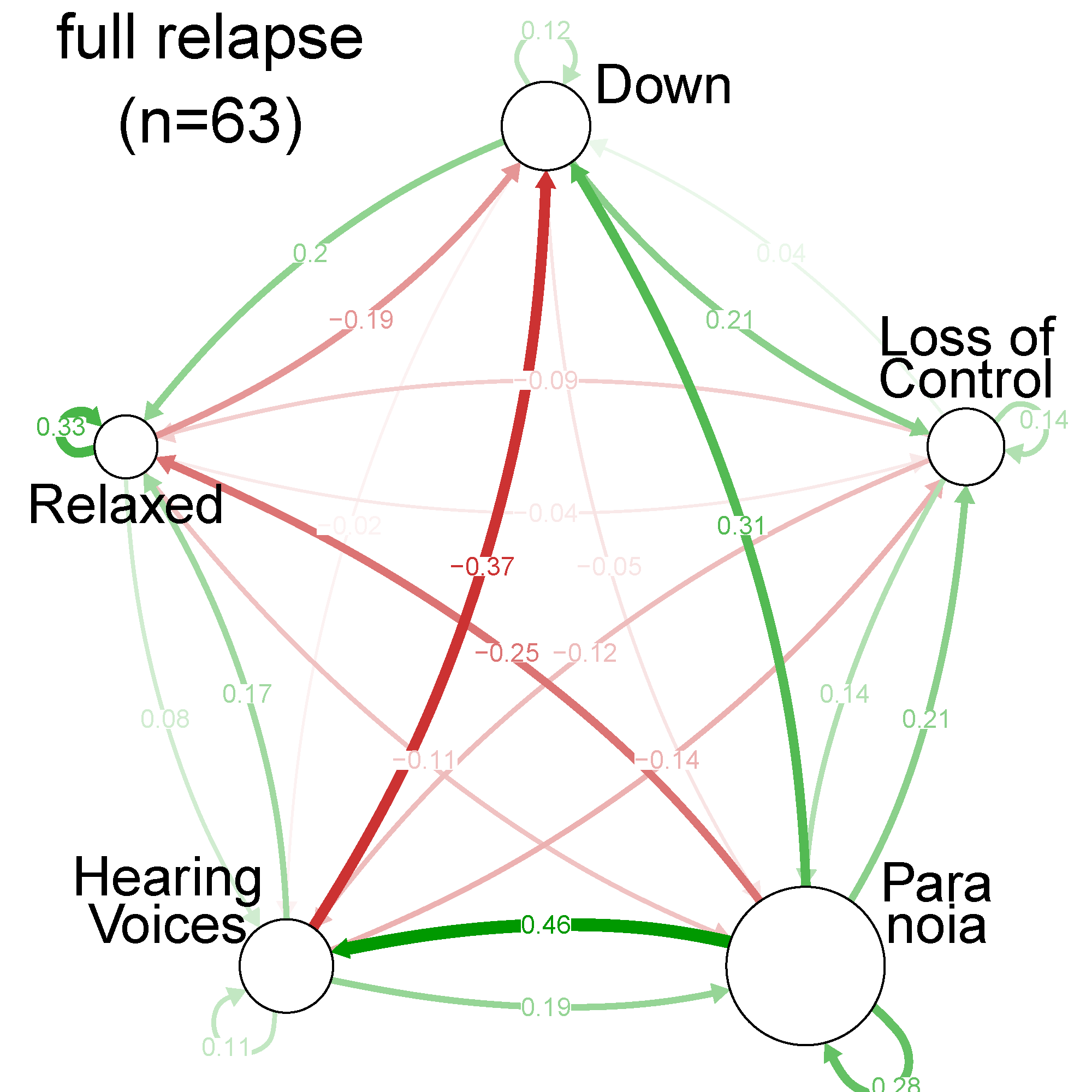

Supplement: S3 Fig — (TIFF) [file pone.0162811.s003.tiff]
