# Supplementary material for: An n=1 Clinical Network Analysis of Symptoms and Treatment in Psychosis
Source: PLoS One. 2016 Sep 19;11(9):e0162811. doi: 10.1371/journal.pone.0162811 (PMC5028060; doi:10.1371/journal.pone.0162811)
Supplement: S1 Table — (DOCX) [file pone.0162811.s004.docx]

**Table S1. Centrality indices per symptom, based on Spearman partial correlation coefficients, for each of the three strata of severity**

|  | Between­ness | Closeness | Inward degree | Outward degree | Node strength |
| --- | --- | --- | --- | --- | --- |
| **Stable state** |  |  |  |  |  |
| ‘Down’ | 3 | 0.029 | 0.76 | 0.80 | 1.57 |
| ‘Loss of control’ | 0 | 0.015 | 0.46 | 0.34 | 0.80 |
| ‘Paranoia’ | 3 | 0.029 | 0.70 | 0.74 | 1.44 |
| ‘Hearing Voices’ | 0 | 0.014 | 0.46 | 0.38 | 0.84 |
| ‘Relaxed’ | 0 | 0.028 | 0.66 | 0.78 | 1.45 |
| **Impending relapse** |  |  |  |  |  |
| ‘Down’ | 0 | 0.042 | 0.65 | 0.83 | 1.48 |
| ‘Loss of control’ | 0 | 0.027 | 0.48 | 0.45 | 0.93 |
| ‘Paranoia’ | 6 | 0.056 | 0.77 | 1.20 | 1.97 |
| ‘Hearing Voices’ | 0 | 0.023 | 0.80 | 0.31 | 1.07 |
| ‘Relaxed’ | 1 | 0.032 | 1.04 | 0.82 | 1.77 |
| **Full Relapse state** |  |  |  |  |  |
| ‘Down’ | 1 | 0.029 | 1.03 | 0.60 | 1.63 |
| ‘Loss of control’ | 2 | 0.027 | 0.74 | 0.53 | 1.27 |
| ‘Paranoia’ | 3 | 0.071 | 0.77 | 1.51 | 2.28 |
| ‘Hearing Voices’ | 0 | 0.048 | 0.80 | 0.98 | 1.78 |
| ‘Relaxed’ | 0 | 0.028 | 1.04 | 0.76 | 1.79 |
